# Virulence and Antimicrobial Resistance in* Campylobacter* spp. from a Peruvian Pediatric Cohort

**DOI:** 10.1155/2017/7848926

**Published:** 2017-10-09

**Authors:** Angela Lluque, Maribel Riveros, Ana Prada, Theresa J. Ochoa, Joaquim Ruiz

**Affiliations:** ^1^Instituto de Medicina Tropical, Universidad Peruana Cayetano Heredia, Lima, Peru; ^2^Department of Epidemiology, School of Public Health, University of Texas Health Science Center at Houston, Houston, TX, USA; ^3^ISGlobal, Barcelona Ctr. Int. Health Res. (CRESIB), Hospital Clínic, Universitat de Barcelona, Barcelona, Spain

## Abstract

The presence of virulence factors (VFs) and mechanisms of quinolones and macrolide resistance was analyzed in* Campylobacter *spp. from a pediatric cohort study in Lima. In 149 isolates (39* Campylobacter jejuni* and 24* Campylobacter coli* from diarrheic cases; 57* C. jejuni* and 29* C. coli* from controls), the presence of the* cdtABC* and* cadF* genes and* iam* marker was established. Nalidixic acid, ciprofloxacin, erythromycin, and azithromycin susceptibilities were established in 115 isolates and tetracycline-susceptibility was established in 100 isolates. The presence of mutations in the* gyrA*,* parC, *and* 23S rRNA* genes was determined. The* cadF* gene and all genes from the* cdtABC* operon were significantly more frequent among* C. jejuni* (*P* < 0.0001); the* iam *marker was more frequent in* C. coli* (*P* < 0.0001). No differences were observed in VFs between cases and controls. Almost all isolates were tetracycline-resistant; nalidixic acid and ciprofloxacin resistance reached levels of 90.4% and 88.7%, respectively. Resistance to macrolides was 13% (*C. jejuni* 4.3%;* C. coli *26.1%). Resistance to ciprofloxacin was related to GyrA Thr86 substitutions, while 13 of 15 macrolide-resistant isolates possessed a* 23S rRNA *mutation (A2075G). Differences in the presence of VFs and alarming levels of resistance to tested antimicrobial agents were observed among* C. jejuni* and* C. coli*.

## 1. Introduction

*Campylobacter *spp. ranks among the most relevant causes of diarrheal illness worldwide, with recent estimations of around 166,000 cases/year, including 31,700 Guillain-Barré Syndromes, which lead to 37,604 deaths and 3,733,822 Disability Adjusted Life Years (DALYs) [1]. In addition, other severe sequelae, such as Miller-Fisher syndrome (a subtype of Guillain-Barré Syndrome), have been described [2, 3]. Although other* Campylobacter *species have clinical relevance,* Campylobacter jejuni *and* Campylobacter coli* have classically been considered the most relevant human pathogens belonging to this genus [2].

Although relatively little is known about the virulence of* Campylobacter* spp., these microorganisms possess different virulence factors (VFs) related to motility, adhesion, invasion, toxin-activity, immune evasion, and iron-uptake, among others [2]. Thus, while factors, like the* cadF* gene or the* iam *locus, are involved in different invasion steps [4, 5] others such as the cytolethal distending toxin, a tripartite toxin encoded in the* cdtA*,* cdtB,* and* cdtC* genes which is also present in other microorganisms [6], block the CDC2 kinase, leading to progressive cellular distension which results in cell death [2].

Diarrhea by* Campylobacter* spp. is usually a self-limited disease which only requires oral rehydration. However, in some cases (immunocompromised patients, long duration of symptoms, and patients with severe complications) the use of antimicrobial agents may be required [7]. Currently, macrolides are the drugs of choice, with fluoroquinolones as second-line drugs quinolones [7]. However, the presence of quinolone-resistant* Campylobacter *spp. isolates is not a novel event [8–10]. Moreover, the development of quinolone resistance during antibiotic treatment has also been reported [7, 11]. In general, the amino acid substitutions in the A subunits (GyrA and ParC) of the DNA-Gyrase and Topoisomerase IV are the most relevant mechanisms of quinolone resistance [12]. In addition, alterations in cytoplasmic quinolone uptake and a series of transferable mechanisms of quinolone resistance (TMQR) also play a role in the increasing levels of quinolone resistance [12, 13]. Interestingly,* Campylobacter* spp. does not possess a Topoisomerase IV, and thus a single amino acid substitution at GyrA may result in high levels of quinolone resistance [12]. The most frequently described amino acid substitution in* Campylobacter *spp. affects positions 86 and 90 of GyrA, with the amino acid change Thr86-Ile being the most widely described [8, 14]. In addition, the relevant role of CmeABC, a resistance-nodulation-cell division (RND) efflux pump, has also been described [15]. Finally, to the best of our knowledge, up to now TMQR has not been described in* Campylobacter *spp.

Regarding macrolides, the isolation of resistant* Campylobacter* spp. is increasingly reported [16, 17], being especially of note in isolates of an animal origin [10, 18]. In both animal and human isolates, macrolide resistance is more frequent in* C. coli* [9, 10, 16, 18]. Macrolides interact with the 50S subunit of the ribosome, inhibiting protein elongation and thus protein synthesis [19]. Alterations at the interaction points of the 23S rRNA, L4, or L22 proteins result in the development of macrolide resistance in a wide range of microorganisms [19]. However, the clinical relevance of mutations in the* 23S rRNA* gene is closely related to the copy number of the gene that each microorganism possesses [19]. Thus, in* Campylobacter *spp., which has 3 copies of the* 23S rRNA* gene, mutations in more than one gene copy results in the development of macrolide resistance [20]. Mutations such as A2074G/T, A2075G, and A2076G (equivalent to A2057G/T, A2058G, and A2059G following* E. coli *numeration) have been described in* Campylobacter *spp., with those affecting A2075 being the most frequently detected [14, 16, 20]. Although L4 and L22 amino acid substitutions, such as the amino acid changes Gly74-Asp in L4 or Ala86-Glu in L22 or the insertions 86::Ala-Arg-Ala-Arg::87 or 98::Thr-Ser-His::99 in L22, have been related to the acquisition of macrolide resistance in* Campylobacter *spp. [14, 21], the role of alterations at L4 and L22 seems to be of less relevance in* Campylobacter *clinical isolates [16, 20]. In fact, it has been described that these alterations may lead to a negative effect on bacterial fitness levels [19]. Additionally, extrusion of macrolides from the bacterial cytoplasm by CmeABC has also been reported [21]. To the best of our knowledge, the* erm*(B) gene, which may be encoded within a transferable multidrug-resistant genomic island, is currently the only transferable mechanism of macrolide resistance (TMMR) described in* Campylobacter *spp. [22].

The aim of this study was to determine the presence of several VFs and the levels and molecular mechanisms of resistance to quinolones and macrolides in a series of* Campylobacter *spp. isolates recovered from children less than 18 months of age, in a periurban area of Lima, Peru.

## 2. Material and Methods

### 2.1. Microorganisms

One hundred forty-nine* Campylobacter *spp. (Supplemental material, available online at https://doi.org/10.1155/2017/7848926) recovered from feces of children less than 18 months old with (63 isolates) and without (86 isolates) diarrhea, during a double-blind controlled trial of bovine lactoferrin for the prevention of diarrhea in children in Lima between January 2008 and May 2011, were included in the study [25]. After initial culture at 42°C in chocolate agar and microaerophilic conditions, followed by* Campylobacter *phenotypic identification (evaluation of colony morphology, Gram staining, and oxidase and catalase determinations), DNA was extracted by direct boiling of 1 colony of each isolate and both DNA and microorganisms were frozen until analysis. A* C. coli* clinical isolate kindly provided by the Instituto Nacional de Salud from Lima (Peru) and* C. jejuni* ATCC 33560,* Escherichia coli *ATCC 25922, and* Staphylococcus aureus* ATCC 25923 were used as control.

### 2.2. Species Determination

*C. coli* and* C. jejuni *were identified by PCR using the primers and conditions previously described (Table 1). The amplified products were analyzed in a 1.5% electrophoresis gel and stained with SYBR Safe (Invitrogen, Eugene, USA). Amplified products were selected at random and sequenced (Macrogen, Seoul, Korea) as quality control.

### 2.3. Virulence Factors

The presence of the* cadF*,* cdtA*,* cdtB,* and* cdtC* genes plus that of the full* cdt* cluster and the* iam* marker was determined by PCR [23] (Table 1).

### 2.4. Antimicrobial Susceptibility

The antimicrobial susceptibility to azithromycin (Azm, 15 *μ*g), erythromycin (Ery, 15 *μ*g), nalidixic acid (Nal, 30 *μ*g), ciprofloxacin (Cip, 5 *μ*g), and tetracycline (Tc, 30 *μ*g) was established by disk diffusion following the EUCAST guidelines in the microorganisms recovered from frozen stock. The EUCAST (Ery, Cip, and Tc) (http://www.eucast.org/fileadmin/src/media/PDFs/EUCAST_files/Breakpoint_tables/v_6.0_Breakpoint_table.pdf) and BSAC (Nal) (http://bsac.org.uk/wp-content/uploads/2012/02/Table-20.pdf) guidelines were used to interpret the obtained diameter. In the absence of established breakpoints, Azm was interpreted according to the following scheme: susceptible ≥ 18 mm and resistant ≤ 17 mm.

### 2.5. Analysis of Mutations in the* gyrA* and* 23S rRNA* Genes

In strains with susceptibility data, the presence of mutations in the* gyrA* and* 23S rRNA *genes was determined by PCR using the primers and conditions previously described (Table 1). In the case of the* gyrA* gene, the DNAs initially obtained for the nongrowing isolates were also included in the study. The amplified products were recovered and purified (PCR Clean-Up System (Promega, Madison, WI)) following the manufacturer's instructions. Both strands of purified products were sequenced (Macrogen, Seoul, Korea).

### 2.6. Statistical Analysis

Fisher's exact test was used to analyze the data. 

## 3. Results

### 3.1. Identification

Of the total strains analyzed, 96 (64.4%) were* C. jejuni* and 53 (35.6%)* C. coli*; of these, 39* C. jejuni* and 24* C. coli* were from diarrheic cases, while 57* C. jejuni* and 29* C. coli* were from healthy controls (Table 2). No differences were found in relation to sex in the prevalence of* C. jejuni *and* C. coli*.

### 3.2. Virulence Factor Analysis

The* cadF* gene was present in all the isolates except 2* C. jejuni* isolates from the control group. The complete* cdtABC* operon was amplified in 87 (58.4%) isolates (85* C. jejuni* and 2* C. coli*) being significantly more frequent among* C. jejuni *(88.7% versus 3.7%) (*P* < 0.001). Regarding the* cdt* genes,* cdtB *was present in 121 isolates (81.2%), while* cdtA* and* cdtC* were present in 102 (67.1%) and 103 (68.7%) isolates, respectively. Independently, all 3 genes were significantly more present in* C. jejuni* than in* C. coli* (*P* < 0.0001) (Table 3). In 1* C. jejuni* full* cdtABC* amplification was achieved; however* cdtA*,* cdtB, *or* cdtC* genes could not be amplified. Similarly 11* C. jejuni* and 4* C. coli* amplify all genes in independent manner, but no PCR product was obtained when the primers for* cdtABC* were used. Regarding the* iam *marker the 3 sequences sought were more frequently detected in* C. coli *than in* C. jejuni *(93.1%, 89.7%, and 96.6% versus 4.0%, 4.0%, and 5.1% for* iam1*,* iam2,* and* iam3,* resp.) (*P* < 0.0001). All 3 sequences were detected concomitantly in the 89.7% of* C. coli* and 4.0% of* C. jejuni *(*P* = 0.0001) (Table 3). No differences in the prevalence of sought VFs were found among isolates from cases and control or sex groups.

### 3.3. Antimicrobial Resistance Levels

The resistance levels to quinolones and macrolides were determined in 115 isolates (69* C. jejuni*, 46* C. coli*) able to grow from frozen stock, while the resistance levels to Tc were also established in 100 out of these isolates.

Regarding quinolones, the results showed almost full concordance (only 2 Nal^R^* C. jejuni* isolates from the diarrhea group were not resistant to Cip) and also extremely high levels of resistance (104 isolates, 90.4% to Nal; 102 isolates, 88.7% to Cip). Likewise, extremely high levels of resistance to Tc were observed (96 isolates, 96.0%). Meanwhile, only 15 (13.0%) isolates showed resistance to both Ery and Azm. All macrolide-resistant microorganisms also showed resistance to the quinolones tested (Table 4).

Analysis by species only showed statistically significant differences in those regarding macrolide resistance. Thus* C. coli *showed higher levels of resistance than* C. jejuni* (12 isolates, 26.1% versus 3 isolates, 4.3%;* P: *0.0012). The significance was also maintained between* C. coli* and* C. jejuni *from the control group (6 isolates, 24% versus 1 isolate, 2.6%; *P* = 0.0119), with borderline significance between* C. jejuni *and* C. coli *from the diarrhea group (*P* = 0.0521) (Table 4).

No association was observed between sex and macrolide or quinolone resistance. No association was found between susceptibility/resistance and a higher or lower presence of the VFs sought.

### 3.4. Analysis of the Mechanisms of Resistance

The analysis of the* gyrA* gene showed the presence of Thr86-Ile amino acid substitutions in the 102 Nal^R^Cip^R^ and in 1 Nal^R^Cip^S^ isolates, while in another* C. jejuni*, Nal^R^Cip^S^, the Thr86-Ala substitution was observed. Additionally, 3* C. jejuni *isolates exhibiting susceptibility to both quinolones also possessed the Thr86-Ile substitution. Meanwhile, for the 34 nongrowing isolates the presence of Thr86-Ile was observed in 28 cases.

Resistance to macrolides was related to the presence of the base change A2075G in 13 out of 15 (86.7%) macrolide-resistant isolates. Interestingly in 2 out of these 13 isolates (both* C. coli*) double peaks were observed, highlighting the presence of mutations in only 1 or 2 of the 3* Campylobacter *spp.* 23S rRNA* gene copies. Finally, 1 of the 2 macrolide-resistant isolates without a mutation in the* 23S rRNA *gene had an Ery halo of 19 mm and an azithromycin halo of 16 mm, while the remaining isolate had no halo to both of the macrolides tested.

## 4. Discussion

### 4.1. Microorganisms

Although a reduction in the burden of diarrhea has been observed in Peru, it has been estimated that in 2015 diarrhea led to 514 deaths in children less than 5 years of age (0.8 deaths/1,000 live births), accounting for 4.9% of deaths in this population (http://apps.who.int/gho/data/node.main.COCD?lang=en). In Peruvian rural zones and in periurban areas of Lima and other cities the lack of adequate sanitation conditions supports the high prevalence of diarrheic diseases. In these areas,* Campylobacter *spp. ranks after enteric viruses and enteropathogenic* E. coli *as etiologic cause of diarrhea [25].

The proportions of* C. jejuni *and* C. coli *in our study are quite different from previous studies performed in this area. Thus, analyzing 4652* Campylobacter *spp. collected between January 2001 and December 2010 the presence of 3856* C. jejuni *(82.9%) and 554* C. coli* (11.9%) was detected together with other* Campylobacter* spp. [17]. Although the spread of a* C. coli *clone in the area may be suggested, there is no clear reason for these differences.

### 4.2. Virulence Factors

Previous studies have shown that almost all* C. jejuni* and* C. coli* possess the* cadF* gene [26, 27]. In this line, our results are as expected. Regarding the presence of 2* cadF* negative* C. jejuni *isolates, although possible insertion inactivation or deletion can not be ruled out, the presence of a polymorphism which might affect PCR-positivity has been previously described [27]. Meanwhile, both in the case of* cdt *and* iam*, the use of different primer sets increased the reliability of PCR results, confirming the presence of significant differences in the carriage of these VFs among* C. coli* and* C. jejuni*.

Although presence of polymorphisms in the primers annealing regions may not be ruled out, while all* C. jejuni *presenting the* cdt* operon possessed the 3 components, a series of* C. coli *were positives for* cdtB* but not for* cdtA* and/or* cdtC.* This is a relevant finding because the lack of either* cdtA* or* cdtC *leads to an impaired production of CDT [28].

Some studies have shown that the IAM region was more frequent in* C. coli *independently of whether it was from children (83.3%) or chicken (100%), being also frequent (54.7%) in* C. jejuni *from chicken but almost absent (1.3%) in those isolated from children [23]. In accordance with this, our results showed that* C. coli* carried the IAM region significantly more frequently than* C. jejuni*.

### 4.3. Antimicrobial Resistance

Symptomatic and asymptomatic* Campylobacter *spp. infections have been involved in reduced weight gain over three-month periods in children [29]. Although symptomatic infections were marginally associated with reduced linear growth over nine-month periods, the severity of the episodes was correlated with greater deficits in both weight gain and linear growth, demonstrating the need for early control of* Campylobacter* infections [29].

A survey performed in Peru between 2001 and 2010 showed an increase in Cip resistance levels of both* C. jejuni* and* C. coli*. In Lima, the levels of Cip resistance were 73.1% and 48.1% for* C. jejuni *and* C. coli,* respectively, in the period 2001–2005, with those values rising to 91.1% and 87.4% in the period 2006–2010, respectively [17]. The most recent values are in accordance with the levels of Cip resistance detected in our isolates.

Similar to that described in other geographical areas [30], our results showed extremely high resistance levels to Tc of 100% among* C. coli* and 90% among* C. jejuni*. Though not used in the treatment of* Campylobacter* infections in young children, this scenario shows that Tc has lost all its utility in the treatment of* Campylobacter* spp. in Peru.

The macrolide resistance was higher in* C. coli* than in* C. jejuni*, similar to what has been observed in other studies [16, 17]. Overall, our macrolide resistance levels were higher than those previously reported in the area of Lima (*C. jejuni* 4.3% versus 1.9%;* C. coli *26.1% versus 5.3% and 5.8%, Ery and Azm, resp.) [17]. In a previous study a significant increase in the* C. coli *Azm resistance over time in Lima was of note [17]. Our data confirm this trend and also show an increase in macrolide resistance among* C. jejuni*. All the macrolide-resistant isolates detected also showed resistance to quinolones, highlighting the need of new antimicrobial agents to treat* Campylobacter *infections.

### 4.4. Mechanisms of Quinolone and Macrolide Resistance

While most microorganisms possess 2 quinolone-targets (DNA-Gyrase and Topoisomerase IV),* Campylobacter *spp. only possess one of the DNA-Gyrases; thus a single target-mutation may lead to both high Nal and Cip resistance levels [8, 12, 31]. The GyrA amino acid change Thr86-Ile has been extensively described in* Campylobacter *spp. [8, 31]. The phenotype Nal^R^Cip^S^ was observed in two* C. jejuni,* in one case related to the Thr86-Ala substitution. It has been observed that the Thr86-Ala substitution leads to increases in the Nal MIC, in some cases just low-bordering the resistance breakpoint, with a lesser effect on the Cip resistance levels [31]. In addition, microorganisms either having the wild type presence of Ala [32] or presenting a mutation leading to the presence of Ala in the equivalent position of GyrA [33] present Nal resistance patterns, albeit usually lower than those produced by other amino acid substitutions, and decreased susceptibility to fluoroquinolones. This may be related to lower alterations in the hydrophobic patterns of the DNA-Gyrase interaction point [12, 32]. The remaining Nal^R^Cip^S^ as well as the 3 Nal^S^Cip^S^ isolates carrying the Thr86-Ile substitution might be explained by an enhanced quinolone uptake that may be due to a malfunction of efflux pumps or to enhanced outer membrane permeability.

The presence of mutations at position A2075 was found in all but 2 macrolide-resistant isolates. In two cases the data suggested the presence of heterozygote isolates, with only one or two mutated* 23S rRNA*. In these cases, as 33–66% of the ribosomes were resistant to the action of the macrolides, the isolates remained resistant to both Azm and Ery. The presence of 2 macrolide-resistant isolates without alterations in the* 23S rRNA* gene may be due to an overexpression of the CmeABC [21, 34]. This option is highly probable in the isolate having a borderline macrolide halo [34], while another explanation, such as the presence of amino acid substitutions in L4 or L22, might be considered in the other case [19]. In addition, the presence of TMMR, such as Erm(B) recently described in* Campylobacter *genus [22] cannot be ruled out.

In summary, the present data demonstrates high levels of Tc and quinolone resistance in both* C. jejuni *and* C. coli* and increasing macrolide resistance among* C. coli*. Moreover, the concomitant resistance to quinolones and macrolides is serious and may lead to the expansion of difficult-to-treat* Campylobacter *spp. isolates. The implementation of control measures which result in a more rational antimicrobial use in human infections, but especially in veterinary settings, is a priority.

## Supplementary Material


*Campylobacter* spp. database.

## Figures and Tables

**Table 1:** Primers and PCR conditions used in the present study.

Target	Description	Primer (5′-3′)	Size (bp)	Ann	Cycles	Ref
Identification						

*C. coli* ^*∗*^		AGGCAAGGGAGCCTTTAATC	364	61	30	[23]
		TATCCCTAT CTACAAATTCGC				
*C. jejuni* ^*∗*^		CATCTTCCCTAGTCAAGCCT	773	61	30	[23]
		AAG ATATGGCACTAGCAAGAC				

Resistance						

*gyrA*	DNA-Gyrase subunit A	ATGATGAGGCAAAAAGAGA	410	55	30	[8]
		TAAACTATGAGGTGGGATGT				
*23S rRNA*		GTAAACGGCGGCCGTAACTA	699	52	35	[24]
		GACCGAACTGTCTCACGACG				

Virulence						

cadF	*Campylobacter* adhesin to fibronectin	TTGAAGGTAATTTAGATATG	400	45	30	[23]
		CTAATACCTAAAGTTGAAAC				
cdtABC	Cytolethal distending Toxin subunits ABC	GGAAATTGGATTTGGGGCTATACT	1215	55	30	[23]
		TTGCACATAACCAAAAGGAAG				
cdtA	Cytolethal distending Toxin subunit A	CCTTGTGATGCAAGCAATC	370	42	30	[23]
		ACACTCCATTTGCTTTCTG				
cdtB	Cytolethal distending Toxin subunit B	GTTAAAATCCCCTGCTATCAACCA	495	42	30	[23]
		GTTGGCACTTGGAATTTGCAAGGC				
cdtC	Cytolethal distending Toxin subunit C	CGATGAGTTAAAACAAAAAGATA	182	42	30	[23]
		TTGGCATTATAGAAAATACAGTT				
iam1	Invasión-associated marker 1	GCGCAAAATATTATCACCC	518	52	30	[23]
		TTCACGACTACTATGCGG				
iam2	Invasion-associated marker 2	GGCGCTTTAGGGAAGCTG	1360	52	30	[23]
		CTTTAAATTGAATCACGGG				
iam3	Invasion-associated marker 3	TGAGGAGCTAAGGGTGCAAA	270	52	30	[23]
		AATACTGATATTTTCCACAT				

bp: base pair; Ann: annealing; Ref: reference. ^*∗*^Primers used in a Multiplex PCR.

**Table 2:** Samples type.

	*n* (%)		
	Diarrhea (*n* = 63)	Asymptomatic control (*n* = 86)	Total (*n* = 149)
*C. jejuni*	39 (61.9)	57 (66.3)	96 (64.4)
*C. coli*	24 (38.1)	29 (33.7)	53 (35.6)
Total	63 (100)	76 (100)	149 (100)

**Table 3:** *Campylobacter* virulence factors.

VF	*C. jejuni*						*C. coli*						*P*	All *Campylobacter*					
	D (39)		C (57)		T (97)		D (24)		C (29)		T (53)			D (63)		C (86)		T (149)	
	*N*	%	*N*	%	*N*	%	*N*	%	*N*	%	*N*	%		*N*	%	*N*	%	*N*	%
*cadF*	39	100.0	55	96.4	95	97.9	24	100.0	29	100.0	53	100.0		63	100	84	97.7	147	98.7
*cdtABC* ^†^	35	89.7	50	87.7	86	88.7^*∗*^	2	8.3	0	0.0	2	3.8^*∗*^	<0.0001	37	58.7	50	59.5	87	58.4
* cdtA*	39	100.0	56	98.2	96	99.0^*∗*^	3	12.5	4	13.8	7	13.2^*∗*^	<0.0001	42	66.7	60	71.4	102	68.5
* cdtB*	39	100.0	56	98.2	96	99.0^*∗*^	11	45.8	15	51.7	26	49.1^*∗*^	<0.0001	50	79.4	71	84.5	121	81.2
* cdtC*	39	100.0	56	98.2	96	99.0^*∗*^	3	12.5	5	17.2	8	15.1^*∗*^	<0.0001	42	66.7	61	72.6	103	69.1
*iam*	3	7.7	1	1.7	4	4.0^*∗*^	21	87.5	26	89.7	47	88.7^*∗*^	<0.0001	24	38.1	27	32.1	51	34.2
* iam1*	3	7.7	1	1.7	4	4.0^*∗*^	23	95.8	27	93.1	50	94.3^*∗*^	<0.0001	26	41.3	28	33.3	54	36.2
* iam2*	3	7.7	1	1.7	4	4.0^*∗*^	21	87.5	26	89.7	47	88.7^*∗*^	<0.0001	24	38.1	27	32.1	51	34.2
* iam3*	4	10.3	1	1.7	5	5.1^*∗*^	23	95.8	28	96.6	51	96.2^*∗*^	<0.0001	27	27.0	29	34.5	56	37.6

D: diarrhea; C: control; T: total; VF: virulence factor; *N*: number; %: percentage. ^*∗*^The presence of significant differences between specific groups. ^†^In 1 *C. jejuni* the *cdtABC* operon was amplified but no individual gene amplification was obtained; similarly in 11 *C. jejuni *and 4 *C. coli* cases the 3 individual genes were amplified, but no amplification for the full *cdtABC* operon was obtained.

**Table 4:** *Campylobacter* antimicrobial resistance levels.

Ab	Antibiotic resistance																	
	*C. jejuni*						*C. coli*						All *Campylobacter*					
	Diarrhea		Control		Total		Diarrhea		Control		Total		Diarrhea		Control		Total	
	*n*/*N*	%	*n*/*N*	%	*n*/*N*	%	*n*/*N*	%	*n*/*N*	%	*n*/*N*	%	*n*/*N*	%	*n*/*N*	%	*n*/*N*	%
Nal	28/30	93.3	34/39	87.2	62/69	89.8	20/21	95.2	22/25	88.0	42/46	91.3	48/51	94.1	56/64	87.5	104/115	90.4
Cip	26/30	86.7	34/39	87.2	60/69	87.0	20/21	95.2	22/25	88.0	42/46	91.3	46/51	90.2	56/64	87.5	102/115	88.7
Ery	2/30	6.7	1/39	2.6^*∗*^	3/69	4.3^‡^	6/21	28.6	6/25	24.0^*∗*^	12/46	26.1^‡^	8/51	15.7	7/64	10.9	15/115	13.0
Azm	2/30	6.7	1/39	2.6^†^	3/69	4.3^#^	6/21	28.6	6/25	24.0^†^	12/46	26.1^#^	8/51	15.7	7/64	10.9	15/115	13.0
Tc	24/27	88.9	32/33	97.0	56/60	93.3	19/19	100.0	21/21	100.0	40/40	100.0	43/46	93.5	53/54	98.1	96/100	96.0

Ab: antibiotic, Nal: nalidixic acid, Cip: ciprofloxacin; Ery: erythromycin; Azm: azithromycin; Tc: tetracycline; *P* < 0.05. Comparison between erythromycin resistance *∗* and azithromycin resistance † of *C. jejuni* and *C. coli* from control groups; *P* < 0.005. Comparison between erythromycin resistance ‡ and azithromycin resistance # of total recovered *C. jejuni* and *C. coli*.
